# Comparative Study of the Flavonoid Content in *Radix Scutellaria* from Different Cultivation Areas in China

**DOI:** 10.1155/2023/3754549

**Published:** 2023-02-10

**Authors:** Yiying Zheng, Shengnan Zhou, Haokang Zhang, Zongyuan Lu, Ruixue Deng, Yihao Feng, Pu Liu

**Affiliations:** ^1^Luoyang Key Laboratory of Natural Products Functional Factor Research and Development, Chemical Engineering & Pharmaceutical College, Henan University of Science and Technology, Luoyang, Henan 471023, China; ^2^College of Horticulture and Plant Protection, Henan University of Science and Technology, Luoyang, Henan 471023, China; ^3^Shanghai Standard Technology Co., Ltd., Pudong District, Shanghai 201314, China

## Abstract

*Scutellariabaicalensis* Georgi, an important perennial herb, is widely distributed and used all over the world. The root of *S. baicalensis* (*Radix Scutellaria*) is rich in flavonoids with a variety of bioactive effects and is widely used in clinic. The different geographical and climatic conditions of different cultivated areas of *S. baicalensis* lead to the differences of the main components in *Radix Scutellaria.* The main objective of this study was to evaluate the difference of flavonoid content in *Radix Scutellaria* from different cultivated areas in China. The mobile phase system, elution gradient, detection wavelength, and other chromatographic conditions for high-performance liquid chromatography-diode array detection (HPLC-DAD) determination of 8 flavonoids in *Radix Scutellaria* were optimized. The contents of flavonoids in 38 samples of *Radix Scutellaria* collected from seven main genuine cultivated areas were determined, and the correlation between the content, cultivated area, and the biological activities of *Radix Scutellaria* was compared. The results implied that baicalin, wogonoside, and baicalein were the three main flavonoids with the highest contents in *Radix Scutellaria*. The content of flavonoids in different cultivated areas was very different, which had significant regionality and was closely related to the natural conditions of various places. The antioxidant and antitumor activities of the extract of *Radix Scutellaria* were closely related to the content of flavonoids, and high contents of baicalin, wogonoside, and baicalein positively improved biological activities.

## 1. Introduction

*Scutellaria baicalensis* Georgi (Chinese skullcap) is a perennial herb belonging to genus *Scutellaria* (Labiatae). The traditional Chinese medicine *Radix Scutellaria* refers to the dry root of *S. baicalensis* and is one of the bulk medicinal materials commonly used all over the world [1, 2]. *Radix Scutellaria* had the effects of clearing away heat and dampness, purging fire and detoxifying, stopping blood gas, and calming fetus and had been used clinically for more than 2000 years in China [1–5]. *Radix Scutellariae* had also played an important role in *Qingfei Paidu Decoction* in the *National Diagnosis Treatment Protocol for COVID-19 in China* [6, 7].

*Radix Scutellaria* is rich in a variety of active ingredients, including flavonoids, glycosides, terpenoids, micronutrient, enzymes, sterols, and organic acids [1, 3, 8]. Flavonoids, typically baicalin, baicalein, wogonin, and wogonoside, are the main active components and the material basis for the pharmacological activities of *Radix Scutellaria* [9–12]. Modern research showed that flavonoids in *Radix Scutellaria* had antioxidant, antibacterial, antiviral, antitumor, anticancer, anti-inflammatory, and other pharmacological effects [1–5, 13–17] and could also improve the levels of monoamine transmitters and brain neurotrophic factors in the brain [18–20].

*S. baicalensis* has strong adaptability to the environment, is widely distributed and commonly used throughout the world, and is also the traditional herbal medicine in China, Mongolia, North Korea, and Japan [2–4]. *S. baicalensis* is geographically widespread in almost all provinces and regions of China, mainly in Hebei, Henan, Gansu, Inner Mongolia, Shaanxi, Shanxi, Shandong, and other places [11, 12, 21–23]. *S. baicalensis* mostly grows in the warm, cool, semi-humid, and semi-arid environment of middle and high mountains or plateau grassland with good sunshine. The diversity of growth environment and the different geographical and climatic conditions of different cultivated areas lead to the differences of the main components in *Radix Scutellaria* [12, 24, 25]. As an important industrial crop, *S. baicalensis* is widely cultivated in China, among which Gansu, Hebei, Henan, Shandong, Shaanxi, Shanxi, and Gansu are the genuine producing areas of *S. baicalensis* [11, 12, 23].

HPLC is a very widely used analytical method with the advantages of simple operation, high efficiency, and high sensitivity and is widely used in a variety of natural active substance content determination [26–28]. In this study, a method for simultaneous determination of 8 main flavonoids in *Radix Scutellaria* was established to evaluate the content differences of main flavonoids in *Radix Scutellaria* collected from different production areas and different planting environments. The principal component analysis (PCA) and hierarchical cluster analysis (HCA) were employed to select the key indexes affecting the quality of *Radix Scutellaria*, and the quality evaluation system of *Radix Scutellaria* was preliminarily established. The study provided research reference for the utilization of *Radix Scutellaria* and the evaluation of the quality of genuine medicinal materials and reference basis for the quality control and evaluation of *S. baicalensis* and related products.

## 2. Materials and Methods

### 2.1. Materials

The *Radix Scutellaria* material (Table 1), collected from different regions of China, was identified as the dry root of the medicinal plant *Scutellaria baicalensis* Georgi by Professor Dingxu Li, School of Agriculture, Henan University of Science and Technology. The geographical and climatic information of different cultivated areas is shown in Table S1.

**Figure 1:**
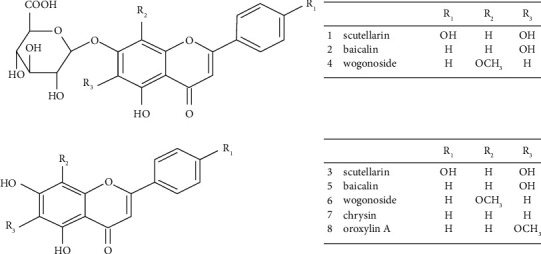
Structural formulas of the 8 flavonoids from *Radix Scutellaria.*

Reference substances (Figure 1), scutellarin (batch number: ST03110120), baicalin (ST01860120), scutellarein (ST06340120), wogonoside (ST08350120), baicalein (ST01870120), wogonin (ST01710120), chrysin (ST00270120), and oroxylin A (ST23660120), were obtained from Shanghai Standard Biotechnology Co. Ltd. with the purity over 98%.

### 2.2. Preparation of the Samples

The samples of *Radix Scutellaria* from different cultivated areas were dried in an oven at 80°C to constant weight and then were crushed with a high-speed pulverizer and sieved with 80 meshes. All samples were kept in sealed bags at room temperature for further research.

### 2.3. Preparation of Working Standard Solutions

The reference flavonoids of scutellarin, baicalin, scutellarein, wogonoside, baicalein, wogonin, chrysin, and oroxylin A were accurately weighed (8–10 mg) and dissolved in an appropriate amount of methanol, and the mixed reference stock solution was prepared with the mass concentration of 0.19 mg·mL^−1^, 0.23 mg·mL^−1^, 0.21 mg·mL^−1^, 0.21 mg·mL^−1^, 0.2 mg·mL^−1^, 0.22 mg·mL^−1^, 0.18 mg·mL^−1^, and 0.19 mg·mL^−1^, respectively. The reference standard solutions were obtained by diluting reference stock solution into six concentration gradients, comprising scutellarin of 19–209 *μ*g·mL^−1^, baicalin of 23–253 *μ*g·mL^−1^, scutellarein of 21–231 *μ*g·mL^−1^, wogonoside of 21–231 *μ*g·mL^−1^, baicalein of 20–220 *μ*g·mL^−1^, wogonin of 22–242 *μ*g·mL^−1^, chrysin of 18–198 *μ*g·mL^−1^, and oroxylin A of 19–209 *μ*g·mL^−1^. The reference standard solutions were filtered with 0.45 *μ*m organic microporous filter membrane, and the filtrate was collected for HPLC analysis.

### 2.4. Preparation of Sample Solution

The sample powder (4.0 g) of *Radix Scutellaria* was accurately weighed and was placed in a 250 mL Soxhlet reflux device and extracted with 100 mL methanol solution in a constant temperature water bath at 85°C for 4 hours under reflux. The extract solution was cooled to room temperature, and then fixed the volume in a 100 mL volumetric flask with methanol. The solution was filtered by 0.45 *μ*m organic microporous filter membrane, and then the filtrate was collected for HPLC analysis.

### 2.5. Apparatus and Chromatographic Conditions

An agilent 1100 high performance liquid chromatograph series system equipped with a quaternionic pump (G1311A), a diode array detector (DAD, G1315A/B), an online vacuum degassing device (G1322A), a column temperature box (G1316A), and an automatic sampler (G1313A) was employed in the determination. The chromatographic column was Agilent Zorbax Eclipse SB-AQ-C18 (250 mm × 4.6 mm, 5 *μ*m). Mobile phase was acetonitrile (A)-0.1% phosphoric acid (B) with the gradient elution of 0 min (A, 30%) ⟶ 5 min (A, 35%) ⟶ 10 min (A, 40%) ⟶ 15 min (A, 50%) ⟶ 22 min (A, 50%) ⟶ 35 min (A, 30%). The flow rate was 1.0 mL·min^−1^ with the detection wavelength of 280 nm, the column temperature of 30°C, and the injection volume of 10 *μ*L.

The separated compounds were identified by comparing the retention time of the test sample with the reference sample. Based on the peak area corresponding to each component, the identified flavonoids were quantitatively analyzed by the external standard method according to the standard correction curve.

(1) $$\begin{array}{c} Y\left(\%\right)=\frac{C_{R}\times A_{x}/A_{R}\times D\times V}{m}\times 100\%\text{,} \end{array}$$

where *Y* is the content of sample; *A*_*x*_ is the peak area of test article; *A*_*R*_ is the peak area of control article; *D* is the dilution multiple of test article; *C*_*R*_ is the concentration of control sample (mg·mL^−1^); *m* is the amount of test sample (g); and *V* is the volume of test sample (mL).

### 2.6. Method Validation Parameters

The contents of 8 flavonoids in *Radix Scutellaria* were determined by HPLC, and the linear relationship and linear range, stability, precision, repeatability, and sample recovery of the established method were evaluated. The calibration curve of each compound was constructed with the mixed reference standard solution containing 8 flavonoids, and the mixed reference standard solution was analyzed with the increase of gradient concentration of the reference standard solutions to investigate the linear range and calculate the regression parameters. The *least squares method* was used to calculate the regression equation between the chromatographic peak area (*y*) and the concentration (*x*) of 8 flavonoids, and the correlation coefficient (*R*^2^) was used to reflect the correlation degree between the peak area and the concentration [12]. The methodological investigation of the determination method was further carried out through the determination of repeatability, stability, precision, and sample recovery according to the method of literature [9, 12, 29]. In order to verify the repeatability of the determination method, six different sample solutions prepared in parallel of *Radix Scutellaria* sample from the same origin (HQ-1) were analyzed, and the contents and relative standard deviation (RSD) values of eight flavonoids were calculated. The tested solution prepared above was stored at room temperature for 0, 2, 4, 8, 12, 16, and 24 hours, the samples were determined, and the contents and RSD values of eight flavonoids were calculated to test the stability of the method. The tested solution prepared above was continuously determined for six times, and contents and RSD values were obtained to test the precision of the method. The accuracy of the method was evaluated through the recovery rate experiment with 0.8 mL, 1.0 mL, and 1.2 mL of mixed control solution containing scutellarin (0.677 mg·mL^−1^), baicalin (1.214 mg·mL^−1^), scutellarein (0.261 mg·mL^−1^), wogonoside (0.704 mg·mL^−1^), baicalein (0.603 mg·mL^−1^), wogonin (0.232 mg·mL^−1^), chrysin (0.107 mg·mL^−1^), and oroxylin A (0.077 mg·mL^−1^), which were added to the known content of *Radix Scutellaria* samples, respectively. The flavonoids were extracted, the contents of the 8 flavonoids were determined under the chromatographic conditions above, and recovery and RSD values were calculated. The limit of detection (LOD) and limit of quantification (LOQ) were determined by continuously diluting the reference standard solution until the signal-to-noise ratio (S/N) was about 3 or 10, respectively.

### 2.7. Determination of Total Flavonoids

The contents of total flavonoids (TFs) in *Radix Scutellaria* from different location of cultivated areas were determined by UV spectrophotometry reported in literature [21]. The samples of *Radix Scutellaria* were extracted by the method described in Section 2.4, and the powder of *Radix Scutellaria* extract was obtained by vacuum concentration and weighed. The dried powder of *Radix Scutellaria* extract (5 mg) was weighed accurately and dissolved in methanol and then transferred into a 25 mL volumetric flask, and then methanol was added to obtain the test solution with a mass concentration of 0.2 mg·mL^−1^. 0.8 ml of 0.1 mol·L^−1^ aluminum trichloride solution and 1 mL of 1 mol·L^−1^ potassium acetate solution were added into the test solution and then mixed well. After bathing in water at 20°C for 20 min, the absorbance of prepared test solution was measured at the wavelength of 415 nm, and methanol was used as the blank control.

The rutin solution of 0.005, 0.01, 0.02, 0.03, 0.04, and 0.05 mg·mL^−1^ was used as abscissa, and the corresponding absorbance was used as ordinate to determine the regression curve. The regression equation was obtained as *y* = 0.06553*x* + 0.00031 (where *x* is absorbance and *y* is the concentration of rutin standard solution, in mg·mL^−1^) with *R*^*2*^ of 0.9997. The total flavonoid content was calculated according to the calibration curve and the method reported in literature [21].

### 2.8. The Bioactive Activities

The antioxidant activities of the total flavonoids extract were evaluated through the inhibitory effects on 2, 2′-azino-bis(3-ethylbenzothiazoline-6-sulfonic acid) (ABTS) and 1,1-Diphenyl-2-picrylhydrazyl (DPPH) radicals according to the method described in literature [30, 31]. The antitumor activity of total flavonoids on tumor cells was determined using the Thiazolyl Blue Tetrazolium Bromide (MTT) method [13].

### 2.9. Data Analysis

In the study, PCA and HCA were employed to select the key indexes affecting the quality of *Radix Scutellaria*, and the quality evaluation system of *Radix Scutellaria* was preliminarily established [32–35]. The study provided research reference for the utilization of *Radix Scutellaria* and the evaluation of the quality of genuine medicinal materials.

Three parallel experiments were conducted in each experimental group, and the experimental results were expressed as mean ± standard deviation (SD). One-way analysis of variance (ANOVA) was used to compare the data between the treatments.

## 3. Results and Discussion

### 3.1. Optimization of Extraction Conditions

As conventional organic solvents, methanol and ethanol were widely used as the solvents in the extraction of flavonoids due to the high polarity of flavonoids in *Radix Scutellaria* [9, 12]. The effects of ultrasonic extraction, Soxhlet extraction, and reflux extraction on the yields of total flavonoids from *S. baicalensis* were compared with those using methanol and ethanol as extraction solvent. The results revealed that the extraction yield of total flavonoids by methanol (10.75%) was significantly higher than that of ethanol (6.88%) under the ultrasonic extraction. The methanol thus was more suitable for the extraction of flavonoids from *Radix Scutellaria* in this study. The yield of total flavonoids by Soxhlet extraction (13.06%) was higher than that by reflux extraction (11.73%) and ultrasonic extraction (10.75%) with methanol used as extraction solvent. It may be that the solubility of flavonoids in solvent has a certain saturation in the extraction process, and the extraction rate of ultrasonic extraction and reflux extraction will not be improved when reaching saturation. While Soxhlet extraction method makes use of the principle of solvent reflux and siphon, the flavonoids can be extracted by pure solvent every time, so the extraction efficiency is higher. The results are consistent with those reported in the literature [36, 37]. Therefore, the Soxhlet extraction method with methanol as the extraction solvent was used to extract flavonoids from samples of *Radix Scutellaria*.

### 3.2. Optimization of Chromatographic Conditions

**Figure 2:**
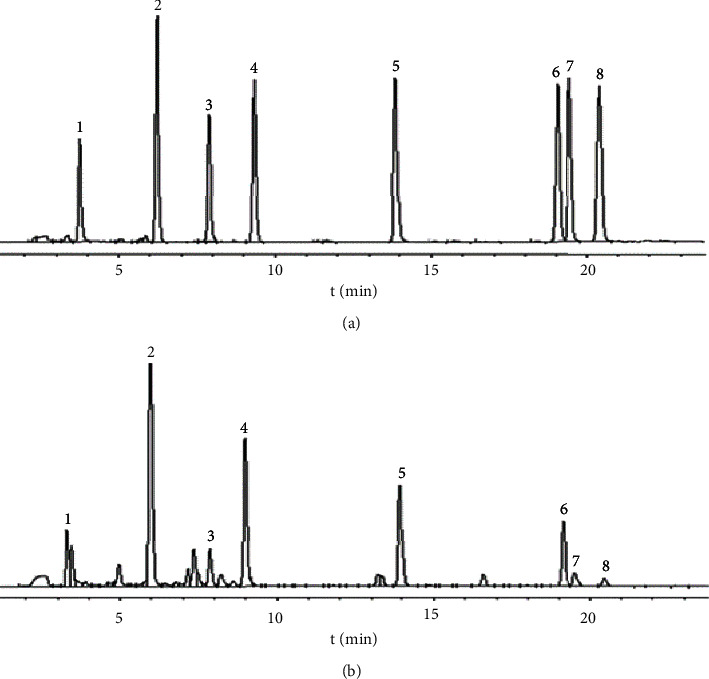
HPLC chromatograms of mixed reference substance (a) and sample (b) (the labeled peaks 1–8 are scutellarin, baicalin, scutellarein, wogonoside, baicalein, wogonin, chrysin, and oroxylin A).

The mobile phase systems, water-acetonitrile-methanol-phosphoric acid (60 : 38 : 30 : 1, V/V/V/V) [9], acetonitrile (A)-0.02% acetic acid (B) [26], methanol (A)-acetonitrile (B)-0.1% formic acid (C) [12], methanol (A)-0.05% formic acid (B) [38], methanol-1% acetic acid (50 : 50 : 1) [39], and acetonitrile (A)-0.1% formic acid (B) [40], had been used formerly for the determination of flavonoids in *Radix Scutellaria*. On the basis of previous research and the multiple comparative experimental studies, the acetonitrile (A)-0.1% phosphoric acid (B) mobile phase system was employed to determine 8 flavonoids at the same time for the effective separation for the determined compounds (Figure 2). Acetonitrile (A)-0.1% phosphoric acid (B) was selected as the mobile phase system, and the optimized gradient elution condition of 0 min (A, 30%) ⟶ 5 min (A, 35%) ⟶ 10 min (A, 40%) ⟶ 15 min (A, 50%) ⟶ 22 min (A, 50%) ⟶ 35 min (A, 30%) was obtained in the study. These chromatographic conditions led to a good separation efficiency with good peak shape and appropriate retention time, and the flavonoids in the sample reached the baseline separation (Figure 2).

In the experiment, the maximum absorption wavelengths of 8 flavonoids were scanned in the wavelength range of 200–400 nm. Among them, scutellarin and scutellarein were well absorbed at 335 nm, and baicalin, baicalein, wogonoside, wogonin, and oroxylin A had the maximum absorption at 280 nm, and the maximum absorption wavelength of chrysin was 250 nm. The results of the selected absorption wavelength were close to the detection wavelengths of 262 nm, 278 nm, and 340 nm reported in the literature [9–12]. The HPLC chromatograms at the wavelengths of 335 nm, 280 nm, and 250 nm were combined, and wavelength of 280 nm was selected in the study for the stable baseline and good resolution (Figure 2).

### 3.3. HPLC Method Validation

The determination method was evaluated through the verification of linearity, precision, repeatability, stability, and sample recovery. The linear range, regression equation, and *R*^*2*^ of 8 flavonoids were obtained as shown in Table 2. The results implied that the method had a good linear relationship and a wide linear range and could be used for the quantitative analysis of flavonoids in *Radix Scutellaria*. Under the same conditions, the same sample was evaluated for 6 times, and the values of RSD of scutellarin, baicalin, scutellarein, baicalein, wogonoside, wogonin, chrysin, and oroxylin A were calculated to be 1.53%, 0.21%, 0.31%, 0.30%, 0.25%, 0.22%, 0.23%, and 0.24%, respectively, indicating that the precision of the instrument was good. The RSD values of the eight components measured after being placed at room temperature for 0, 2, 4, 8, 12, 16, and 24 hours were 1.54%, 0.84%, 0.77%, 0.73%, 0.62%, 0.93%, 0.98%, and 0.92%, respectively, which revealed that the prepared solution has good stability within 24 hours. Six sample solutions were extracted in parallel under the same conditions for determination, and the RSD values were 1.41%, 0.65%, 0.77%, 0.75%, 0.71%, 0.76%, 0.96%, and 0.99%, respectively, and the results implied that the method has good repeatability. The average recovery of the 8 compounds was 99.066–100.433% with the RSD of 0.619–1.763%, which indicated that the method had good sample recovery (Table S2). The LOD and LOQ were determined, and the results are shown in Table 2.

The results suggested that the precision, stability, repeatability, and sample recovery of this method met the analysis requirements, and the method had the characteristics of fast, accurate, reliable, and strong specificity. Therefore, the method was suitable for the content determination of flavonoids in *Radix Scutellaria* and could also provide a scientific basis for the quality evaluation and effective quality control of *Radix Scutellaria*.

### 3.4. Method Application

An HPLC method was established for the simultaneous determination of 8 main flavonoids in the extracts of 38 *Radix Scutellaria* samples from seven cultivated areas. The chromatograms of the mixed control and representative sample at the detection wavelength of 280 nm are shown in Figure 2. The results revealed that the eight flavonoids were completely separated from each other and the baseline was stable. The retention time of all the compounds to be analyzed was within 25 min, and the test time was much smaller than that (30–50 minutes) reported in the literature [9–12], indicating that this method can significantly shorten the test time. The monomer contents of 8 flavonoids in samples from different cultivated areas are shown in Table 3. The flavonoids were confirmed according to the comparison of retention time results between the samples and the reference substance.

The results implied that 8 flavonoids were generally found in 38 samples from different cultivated areas. There were significant differences in the contents of 8 flavonoids in *Radix Scutellaria* from different cultivated areas (*P* < 0.01). Baicalin was the highest among the eight flavonoids in the samples with the content from 46.972 mg·g^−1^ to 194.956 mg·g^−1^, accounting for over 77% of the total content of the eight flavonoids (TCEF) in the samples. Compounds wogonoside and baicalein were another two flavonoids with high content in *Radix Scutellaria* with the contents of 2.378–11.82 mg·g^−1^ and 0.993–16.609 mg·g^−1^, respectively. Compounds chrysin (0.438–2.641 mg·g^−1^) and oroxylin A (0.027–2.652 mg·g^−1^) were the two flavonoids with relatively low content in *Radix Scutellaria*. Scutellarin, scutellarein, and wogonin were three flavonoids with medium contents of 1.057–11.949 mg·g^−1^, 1.221–4.967 mg·g^−1^, and 0.118–5.181 mg·g^−1^ in different cultivated areas, respectively. The total content of the TCEF in the samples ranged from 53.330 mg·g^−1^ to 244.094 mg·g^−1^.

Among all the samples, HQ-5 (194.956 mg·g^−1^), HQ-33 (181.392 mg·g^−1^), and HQ-16 (180.185 mg·g^−1^) had the highest content of baicalin, while HQ-20 (46.972 mg·g^−1^) and HQ-35 (52.686 mg·g^−1^) had the lowest baicalin content. The content of baicalin in HQ-5 sample was over 4 times than that of HQ-20 sample, indicating that the content of baicalin in samples from different cultivated areas varied greatly. In addition to baicalin, the contents of scutellarin, scutellarein, wogonoside, baicalein, chrysin, oroxylin A, and TCFF in HQ-20 sample were the lowest in all samples, only with the contents of 1.057 mg·g^−1^, 1.221 mg·g^−1^, 2.378 mg·g^−1^, 0.993 mg·g^−1^, 0.438 mg·g^−1^, 0.027 mg·g^−1^, and 53.330 mg·g^−1^, respectively, which revealed that the sample might be the worst quality. The content of scutellarin in HQ-37 sample (11.949 mg·g^−1^) was the highest in all samples, while the contents of wogonin and oroxylin A were at low level only with the content of 0.560 mg·g^−1^ and 0.526 mg·g^−1^, respectively. In HQ-30 sample, the content of scutellarein (4.967 mg·g^−1^) was the highest in all the samples. Among the samples from all cultivated areas, baicalin, wogonoside, baicalein, and TCFF in HQ-5 sample were the highest with contents of 19.496 mg·g^−1^, 11.82 mg·g^−1^, 16.609 mg·g^−1^, and 244.094 mg·g^−1^, respectively. The contents of scutellarin (7.281 mg·g^−1^), scutellarein (3.752 mg·g^−1^), wogonin (4.993 mg·g^−1^), chrysin (2.641 mg·g^−1^), and oroxylin A (2.041 mg·g^−1^) were all at high level, which implied that the quality of the sample from this cultivated area was the best.

The above results implied that the main active components of flavonoids in *Radix Scutellaria* were baicalin, wogonoside, baicalein, wogonin, and scutellarin, which were consistent with the research results reported in the literature [10, 12]. The contents of these active components could be used to evaluate the quality of medicinal plant *S. baicalensis*. There were significant differences in the content of flavonoids in different cultivated areas, which revealed that the growth environment had an important impact on the content of flavonoids in *Radix Scutellaria* [11, 24, 25].

Baicalin, wogonoside, and baicalein had good antibacterial property [41–43], which was consistent with the results that *Radix Scutellariae* had been used to treat antibacterial diseases in clinic [44, 45]. Baicalin and baicalein also had potential as therapeutic or supplementary agents for the treatment of breast cancer [46], and the anticancer properties of *Radix Scutellariae* could be attributed to its high content of wogonin, baicalein, and baicalin [3]. A study conducted by Zhang also revealed that baicalin might be one of the main components of *Radix Scutellariae* in the treatment of fetal irritability [47]. It was found that baicalin and baicalein were also the main active substances of traditional Chinese medicine *Radix Scutellariae* with good liver protection and inhibition of liver injury [48]. Baicalein and baicalin were also considered the main material basis of *Radix Scutellariae* for the treatment of the infection of the upper respiratory tract and to cure hyperactivity cough [49–51]. The above studies had confirmed that baicalin and baicalein were the main active substances of *Radix Scutellariae* with a variety of bioactive effects.

The average content of the eight flavonoids in different planting areas is shown in Table 4. It could be seen from the Table 4 that the contents of flavonoids in the samples from seven main genuine producing areas were significantly different (*P* < 0.01). The average contents of baicalin (163.999 mg·g^−1^), wogonoside (10.014 mg·g^−1^), baicalein (15.449 mg·g^−1^), wogonin (4.638 mg·g^−1^), chrysin (2.340 mg·g^−1^), and oroxylin A (1.875 mg·g^−1^) in Gansu cultivated area were all higher than those in other six genuine producing areas, and the content of scutellarin (6.695 mg·g^−1^) and scutellarein (3.072 mg·g^−1^) was at a high level, which resulted in the highest content of TCEF (208.081 mg·g^−1^) and TF (10.778%). The results implied that the quality of *S. baicalensis* in Gansu planting cultivated area was the best as the content of main flavonoids was significantly higher than that in other planting cultivated areas. The cultivated areas of Shaanxi were characterized by the highest content of scutellarin (7.066 mg·g^−1^) and the lowest content of wogonoside (6.314 mg·g^−1^). Compared with that in other planting areas, the samples of Henan cultivated area had the highest content of scutellarein (3.662 mg·g^−1^), and the content of baicalin (158.801 mg·g^−1^), wogonoside (9.223 mg·g^−1^), wogonin (2.816 mg·g^−1^), and oroxylin A (1.280 mg·g^−1^) in Henan cultivated area was only lower than that in Gansu cultivated area, while it was higher than that in the other five cultivated areas, which led to the content of TCEF (190.975 mg·g^−1^) only lower than that in Gansu and higher than that in the samples from other cultivated areas. The cultivated area in Inner Mongolia was characterized by the lowest content of baicalin (131.090 mg·g^−1^), baicalein (5.747 mg·g^−1^), and wogonin (1.801 mg·g^−1^), and the content of wogonoside (6.410 mg·g^−1^) was also at a low level, which resulted in the lowest content of TCEF (156.590 mg·g^−1^) in the samples of cultivated area in Inner Mongolia. The contents of chrysin (1.289 mg·g^−1^), scutellarein (2.446 mg·g^−1^), and oroxylin A (0.876 mg·g^−1^) in the samples from Hebei cultivated areas were the lowest among all cultivated areas. At the same time, the contents of scutellarin, wogonoside, and baicalein were also at a low level, which was the main reason that TCEF and TF were at a low level in the samples of Hebei cultivated areas.

It was traditionally believed that the genuine producing areas of *S. baicalensis* were in Shandong, Henan, Gansu, Shanxi, Hebei, Inner Mongolia, and other places [23]. In this study, the evaluation content of flavonoids in Shanxi, Hebei, and Inner Mongolia Autonomous Region was not high, which was consistent with the research results reported in the literature [11, 22] but different from the research which believed that the content of active components in *Radix Scutellariae* in Hebei and Inner Mongolia was the highest [21], which might be due to different sampling ranges and other reasons. From the experimental results, the quality of *Radix Scutellariae* in the traditional genuine producing areas was not necessarily the best. The quality of *Radix Scutellariae* from different cultivated areas could be improved by improving germplasm resources and changing cultivation methods.

The studies of the effects of different elevations and different lighting conditions (shade slope and sunny slope) on the contents of flavonoids revealed that high altitude and sunny slope were conducive to increasing the accumulation of total flavonoids in *Radix Scutellariae* [24]. The effects of environmental factors on photosynthetic physiology and flavonoid constituent of Radix Scutellariae also suggested that photosynthetic active radiation and soil water content were important environmental factors impacting on photosynthesis of S. baicalensis, and soil water content, relative humidity, and atmospheric CO2 concentration were important environmental factors impacting on baicalin content, and photosynthetic active radiation, atmospheric pressure, and atmospheric temperature were important environmental factors impacting on baicalin content [25]. The high average altitude, mild and humid climate, and strong light intensity might be the reason for the high content of flavonoids in *Radix Scutellariae* in Tanchang County.

As a bulk medicinal material and a widely used traditional Chinese medicine, *S. baicalensis* had a large planting scale in China, with the characteristics of large planting area and wide range. The difference of climate, geographical environment, and other external factors would affect the active components in *Radix Scutellariae*. Therefore, the systematic determination of the content of main flavonoids will provide a guarantee for the quality evaluation of medicinal materials in different cultivated areas.

### 3.5. Principal Component Analysis

Based on the above content determination results, scutellarin (*X*1), baicalin (*X*2), wogonoside (*X*3), baicalein (*X*4), and total flavonoids (*X*5) were selected as indicators for PCA. One-way ANOVA was used to compare the data between the treatments, and Bartlett's test of sphericity was used to detect the correlation between variables [32, 35]. All statistical analyses were performed at 95% confidence level using SPSS 26.0 software. From the analysis results (Table 5), it could be seen that the value of the Kaiser–Meyer–Olkin (KMO) measure of sampling adequacy (0.587) in this example was greater than 0.5, indicating that PCA could be used to evaluate the results [32, 35]. The significance of Bartlett's test of sphericity (0.001) was less than 0.01, which revealed that the correlation between variables was very significant, and factor analysis could be carried out in the study [32, 35].

The results of PCA (Table 6) of the above five components (*X*1–*X*5) showed that the characteristic values of the first two principal components (*X*1 and *X*2) were greater than 1, and the contribution rate of cumulative variance was 93.028%, and the characteristic value of the first principal component was 3.574 and the contribution rate was 71.477%, representing 71.477% of all information, which implied that scutellarin was a main factor closely related to the origin of *Radix Scutellariae*. The above results implied that these two factors have a high degree of explanation for the whole result [33]. The two principal components could be used to replace the above five specific contents to evaluate the quality of *Radix Scutellariae* from different planting areas.

The principal component matrix was employed for the selection of the principal components, the original data were normalized and the variables were saved, and the PCA model according to the principal component matrix was constructed (Table 7). The principal component model was calculated as follows:

(2) $$\begin{array}{c} F=0.768F_{1}+0.232F_{2}\text{,}\\ F_{1}=0.228X_{1}+0.479X_{2}+0.478X_{3}+0.484X_{4}+0.506X_{5}\text{,}\\ F_{2}=0.459X_{1}-0.182X_{2}-0.203X_{3}+0.034X_{4}+0.124X_{5}\text{.} \end{array}$$

According to the principal component model, the principal component scores of seven planting areas were obtained (Table 8). The results implied that the top three scores in the comprehensive evaluation were 2.963 in Gansu, 0.341 in Shandong, and 0.307 in Henan, indicating that the quality of *Radix Scutellariae* in Gansu planting area was the best and could be used as genuine medicinal material, followed by Shandong and Henan.

### 3.6. Hierarchical Cluster Analysis of the Samples

**Figure 3:**
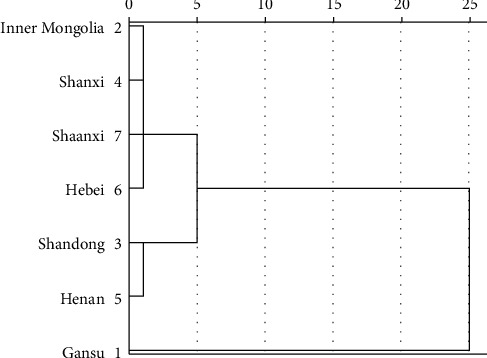
Clustering plot of *Radix Scutellariae* samples.

Taking the content of 8 flavonoids and total flavonoids in *Radix Scutellariae* as indicators, the clustering analysis of *Radix Scutellariae* from different cultivated areas was carried out by the clustering method of square Euclidean distance coefficient and centroid clustering (Figure 3). The results revealed that the seven cultivated areas could be divided into three groups: group I was Shandong and Henan; group II was Inner Mongolia, Hebei, Shaanxi, and Shanxi; and group III was Gansu. There were similarities and differences in the content of flavonoids in *Radix Scutellariae* from different cultivated areas. The contents of baicalin, wogonoside, and baicalein in group III were significantly higher than those in group I and group II. The contents of scutellarin, scutellarein, and chrysin in group I were higher than those in group II. Due to the high content of baicalin, wogonoside, and baicalein, the quality of *Radix Scutellariae* in Gansu Province was the best. Shandong and Henan were clustered together earlier, which might be related to the difference of their growth environment and the small differentiation of population diversity, and results were consistent with the research results reported in the literature [11].

The cluster analysis method reflected the diversity differentiation of *S. baicalensis* in different cultivated areas, and the different cultivated areas would make the chemical composition and content of active components of *Radix Scutellariae* different. It also revealed that the quality of *Radix Scutellariae* was affected by geographical location and environmental factors, which might be related to the superposition of precipitation, atmospheric temperature, sunshine duration, and soil properties [11, 24, 25].

### 3.7. The Bioactive Activities

The antioxidant activities of the total flavonoids extract were evaluated through the inhibitory effects on ABTS and DPPH radicals, and the antitumor activity of total flavonoids on HepG2 was also determined using the MTT method, and the IC_50_ values are shown in Table 9. The IC_50_ values of oroxylin A (67.389 ± 3.14 *μ*g·mL^−1^), scutellarin (65.913 ± 4.22 *μ*g·mL^−1^), wogonin (89.005 ± 5.62 *μ*g·mL^−1^), wogonoside (133.469 ± 8.24 *μ*g·mL^−1^), baicalein (69.608 ± 4.01 *μ*g·mL^−1^), chrysin (123.324 ± 7.11 *μ*g·mL^−1^), and baicalin (70.902 ± 4.09 *μ*g·mL^−1^) on HepG2 were also determined in the study.

The results suggested that the flavonoid extracts of *Radix Scutellariae* from different cultivated areas had certain effects of scavenging free radicals and inhibitory activity on cancer cells, but the activity varied greatly in different cultivated areas. The sample from Gansu Province had the strongest scavenging effect on DPPH and ABTS free radicals, as well as the inhibitory effect on cancer cell HepG2, which was closely related to the high content of flavonoids in the samples. From the inhibitory activity of flavonoids on cancer cells, although the compounds scutellarin (65.913 ± 4.22 *μ*g·mL^−1^) and oroxylin A (67.389 ± 3.14 *μ*g·mL^−1^) had greater inhibitory effect on cancer cell HepG2 than that of other compounds, the effects of the two compounds on the anticancer activity of the samples from corresponding places were weak because of the relatively low contents in the samples. Baicalein and baicalin were the main flavonoids in *Radix Scutellariae*, and the high antitumor activities of baicalein (69.608 ± 4.01 *μ*g·mL^−1^) and baicalin (70.902 ± 4.09 *μ*g·mL^−1^) along with the high content led to strong anticancer activity in Henan and Shanxi samples. From the content determination results, baicalin, baicalein, wogonoside, and wogonin were the main flavonoids with high content in *Radix Scutellariae*. It was found that the order of free radical elimination activity of these four flavonoids was baicalin > baicalein > wogonin > wogonoside [52]. The higher content of baicalin was main reason that Henan samples had strong inhibitory effects on DPPH free radicals. Although the content of flavonoids was not high, Hebei sample had a strong inhibitory effect on DPPH free radical with the low IC_50_ values (39.022 ± 0.276 of ABTS and 125.602 ± 0.539 of DPPH), which implied that there might be other flavonoids with high antioxidant activity in the samples.

However, based on the results of activity determination, the activities of flavonoids extract did not have a good positive correlation with the total content of flavonoids in *Radix Scutellariae*, and higher total flavonoid content did not mean a higher bioactivity. It implied that the bioactivities of *Radix Scutellariae* were related to the content of active flavonoids and the bioactivity of the flavonoids.

## 4. Conclusions

In this study, a method for the simultaneous determination of 8 main flavonoids in *Radix Scutellariae* from different genuine producing areas was established for the first time, and the chromatographic conditions were optimized. The chromatographic conditions with good resolution and appropriate retention time were obtained. The results suggested that flavonoids in *Radix Scutellariae* from different cultivated areas varied greatly, and baicalin, wogonoside, baicalein, wogonin, and scutellarin were the main flavonoids in *Radix Scutellariae*, and the content of baicalin was the highest, accounting for more than 77% of the total flavonoids. The content of flavonoids in *Radix Scutellariae* planted in Gansu cultivated areas was the highest with the content of baicalein, baicalin, wogonin, chrysin, wogonoside, and oroxylin A, and TCEF and TF were much higher than those in other cultivated areas. It implied that the content of flavonoids in *Radix Scutellariae* of Gansu was high in the cultivated areas, which implied that the quality of *Radix Scutellariae* might be good. In addition, the content of flavonoids in *Radix Scutellariae* in Henan and Shandong cultivated areas was also at high level. The results of PCA revealed that Gansu, Shandong, and Henan were the three producing areas with the highest content of flavonoids and the best quality. The results of HCA also confirmed that *S. baicalensis* planted in Gansu cultivated areas had higher content of flavonoids than those of other cultivated areas. The antioxidant and anticancer activities of the extract of *Radix Scutellaria* were closely related to the content of flavonoids, and high contents of baicalin, wogonoside, and baicalein were helpful to improve biological activities. The results implied that there were great differences in the content of flavonoids in samples from different genuine producing areas of *S. baicalensis*. This study had good guiding significance for the quality evaluation of *Radix Scutellariae*.

## Figures and Tables

**Table 1:** Collection information and numbers of *Radix Scutellaria* samples from different origins.

Samples	Cultivated areas	Location
HQ-1	Gansu Province (A)	Min County
HQ-2		Weiyuan County
HQ-3		Zhang County
HQ-4		Hexi District
HQ-5		Tanchang County

HQ-6	Inner Mongolia Autonomous Region (B)	Hohhot
HQ-7		Ningcheng County
HQ-8		Songshan District
HQ-9		Songshan District

HQ-10	Shandong Province (C)	Juancheng County
HQ-11		Juancheng County
HQ-12		Laiwu District
HQ-13		Yishui County
HQ-14		Hedong District
HQ-15		Ju County
HQ-16		Ju County
HQ-17		Yinan County
HQ-18		Wulian County
HQ-19		Zhucheng city

HQ-20	Shanxi Province (D)	Quwo County
HQ-21		Quwo County
HQ-22		Jiang County
HQ-23		Xiangfen County
HQ-24		Wenxi County
HQ-25		Wenxi County
HQ-26		Xinjiang County

HQ-27		Yichuan County
HQ-28	Henan Province (E)	Yichuan County
HQ-29		Yiyang County
HQ-30		Yiyang County
HQ-31		Hui County
HQ-32	Heibei Province (F)	Anguo city
HQ-33		Anguo city
HQ-34		Weichang County

HQ-35	Shaanxi Province (G)	Heyang County
HQ-36		Heyang County
HQ-37		Shangzhou District
HQ-38		Tongguan County

**Table 2:** Validation parameters of the developed RP-HPLC/DAD method.

Compounds	Regressive equation	*R* ^*2*^	Linear range (*μ*g·mL^−1^)	LOD (*μ*g·mL^−1^)	LOQ (*μ*g·mL^−1^)	Recovery (%) (RSD %)
Scutellarin	*y* = 12.131*x* *+* 173.310	0.9994	19.00–209.00	0.558	1.859	99.1 (1.34)
Baicalin	*y* = 22.262*x* *+* 3.827	0.9998	23.00–253.00	0.795	2.650	99.5 (0.62)
Scutellarein	*y* = 40.394*x* − 13.768	0.9999	21.00–231.00	0.614	2.048	99.1 (1.50)
Wogonoside	*y* = 21.478*x* − 8.961	0.9996	21.00–231.00	0.656	2.187	100.0 (1.34)
Baicalein	*y* = 24.628*x* − 18.258	0.9998	20.00–220.00	0.581	1.937	100.4 (1.19)
Wogonin	*y* = 39.549*x* − 10.583	0.9997	22.00–242.00	0.646	2.154	99.2 (1.76)
Chrysin	*y* = 49.741*x* − 11.652	0.9999	18.00–198.00	0.529	1.765	99.2 (1.44)
Oroxylin A	*y* = 62.627*x* − 15.032	0.9995	19.00–209.00	0.508	1.692	99.7 (1.26)

**Table 3:** Content of main flavonoids (mg·g^−1^) and the total flavonoids (TFs, %) in the sample.

Sample	Scutellarin	Baicalin	Scutellarein	Wogonoside	Baicalein	Wogonin	Chrysin	Oroxylin A	TCEF^*∗*^	TF
HQ-1	6.038 ± 0.082	160.114 ± 1.093	2.653 ± 0.231	9.420 ± 0.730	15.001 ± 1.095	4.574 ± 0.420	2.291 ± 0.187	1.904 ± 0.173	201.995	9.386
HQ-2	6.897 ± 1.063	150.800 ± 0.568	2.943 ± 0.411	9.714 ± 1.271	15.590 ± 0.874	4.840 ± 0.536	2.388 ± 0.046	1.792 ± 0.025	194.963	10.963
HQ-3	6.280 ± 0.392	147.888 ± 0.485	2.753 ± 0.177	9.068 ± 0.449	15.226 ± 0.289	4.337 ± 0.238	2.420 ± 0.111	1.807 ± 0.093	189.778	9.314
HQ-4	6.979 ± 0.416	166.238 ± 0.640	3.257 ± 0.034	10.047 ± 0.381	14.820 ± 0.469	4.446 ± 0.269	1.961 ± 0.131	1.828 ± 0.109	209.575	11.285
HQ-5	7.281 ± 0.048	194.956 ± 0.941	3.752 ± 0.041	11.82 ± 0.107	16.609 ± 0.078	4.993 ± 0.053	2.641 ± 0.058	2.041 ± 0.031	244.094	12.952
HQ-6	7.856 ± 0.016	159.858 ± 0.661	3.723 ± 0.051	9.038 ± 0.243	6.168 ± 0.033	1.444 ± 0.011	1.973 ± 0.016	1.071 ± 0.012	191.129	7.229
HQ-7	2.882 ± 0.037	108.092 ± 0.396	2.498 ± 0.056	5.244 ± 0.073	4.609 ± 0.043	1.011 ± 0.018	1.601 ± 0.002	0.799 ± 0.007	126.737	4.052
HQ-8	6.029 ± 0.330	114.696 ± 0.283	2.480 ± 0.200	5.332 ± 0.503	4.758 ± 0.169	1.694 ± 0.070	1.260 ± 0.040	1.310 ± 0.038	137.559	4.366
HQ-9	6.713 ± 0.336	141.735 ± 0.409	3.132 ± 0.105	6.027 ± 0.189	7.452 ± 0.269	3.054 ± 0.134	1.397 ± 0.049	1.424 ± 0.056	170.933	5.733
HQ-10	2.867 ± 0.054	171.291 ± 0.943	2.766 ± 0.143	11.572 ± 0.752	2.992 ± 0.039	0.822 ± 0.028	0.700 ± 0.001	0.227 ± 0.003	193.237	4.527
HQ-11	5.140 ± 0.065	141.157 ± 0.097	2.152 ± 0.093	7.869 ± 0.640	10.809 ± 0.285	3.449 ± 0.076	1.113 ± 0.083	0.816 ± 0.075	172.505	6.305
HQ-12	8.582 ± 0.064	162.409 ± 0.180	4.270 ± 0.095	9.756 ± 0.232	6.632 ± 0.168	1.337 ± 0.009	2.276 ± 0.064	1.047 ± 0.011	196.310	7.804
HQ-13	4.193 ± 0.457	160.339 ± 1.190	3.479 ± 0.020	8.447 ± 0.532	5.854 ± 0.380	1.164 ± 0.052	2.380 ± 0.133	0.987 ± 0.066	186.843	5.648
HQ-14	6.677 ± 0.267	144.045 ± 0.458	3.898 ± 0.036	8.876 ± 0.205	3.888 ± 0.112	0.621 ± 0.108	1.865 ± 0.214	0.540 ± 0.006	170.409	5.155
HQ-15	3.293 ± 0.320	84.129 ± 0.490	1.398 ± 0.096	4.640 ± 0.168	2.584 ± 0.026	0.648 ± 0.043	1.017 ± 0.247	0.378 ± 0.131	98.088	2.867
HQ-16	8.405 ± 0.079	180.185 ± 1.196	3.910 ± 0.254	10.991 ± 0.623	10.794 ± 0.345	3.509 ± 0.215	2.091 ± 0.123	1.274 ± 0.077	221.159	9.735
HQ-17	6.731 ± 0.266	133.749 ± 0.250	3.133 ± 0.028	7.381 ± 0.030	5.300 ± 0.080	1.168 ± 0.043	2.228 ± 0.081	0.974 ± 0.030	160.665	5.134
HQ-18	6.977 ± 0.268	161.894 ± 0.555	3.325 ± 0.304	9.798 ± 1.416	16.066 ± 0.195	5.012 ± 0.551	1.991 ± 0.213	1.745 ± 0.189	206.808	12.159
HQ-19	7.246 ± 0.180	154.120 ± 1.237	3.102 ± 0.207	7.965 ± 0.127	15.030 ± 0.004	5.103 ± 0.073	2.506 ± 0.028	1.319 ± 0.021	196.391	9.318
HQ-20	1.057 ± 0.292	46.972 ± 0.062	1.221 ± 0.052	2.378 ± 0.128	0.993 ± 0.077	0.245 ± 0.007	0.438 ± 0.031	0.027 ± 0.016	53.330	1.988
HQ-21	5.414 ± 0.246	138.679 ± 1.45	3.436 ± 0.640	6.880 ± 0.742	9.930 ± 0.846	3.018 ± 0.132	1.398 ± 0.080	1.197 ± 0.078	169.951	6.119
HQ-22	6.756 ± 0.101	160.256 ± 0.959	3.517 ± 0.190	9.118 ± 0.401	6.594 ± 0.365	2.005 ± 0.090	1.095 ± 0.004	0.941 ± 0.023	190.282	6.619
HQ-23	5.804 ± 0.187	155.457 ± 0.818	3.183 ± 0.060	6.757 ± 0.352	8.428 ± 0.170	2.409 ± 0.052	1.430 ± 0.024	1.298 ± 0.028	184.765	6.044
HQ-24	3.395 ± 0.061	105.854 ± 0.184	2.178 ± 0.048	5.708 ± 0.228	12.924 ± 0.999	3.382 ± 0.066	1.712 ± 0.172	1.356 ± 0.157	136.509	5.491
HQ-25	8.065 ± 0.104	160.150 ± 1.229	1.334 ± 0.108	9.383 ± 1.036	13.494 ± 0.609	4.253 ± 0.118	2.029 ± 0.051	1.775 ± 0.047	200.481	8.279
HQ-26	5.707 ± 0.281	157.037 ± 0.820	3.311 ± 0.101	7.220 ± 0.178	10.635 ± 0.487	3.438 ± 0.223	1.570 ± 0.091	1.327 ± 0.080	190.245	7.686
HQ-27	4.148 ± 0.060	178.953 ± 0.874	2.608 ± 0.047	11.332 ± 0.141	6.150 ± 0.396	2.478 ± 0.019	1.476 ± 0.028	0.531 ± 0.003	207.676	6.744
HQ-28	5.985 ± 0.496	164.129 ± 1.142	4.048 ± 0.199	8.404 ± 0.262	10.872 ± 0.347	3.214 ± 0.223	1.210 ± 0.181	1.441 ± 0.095	199.303	8.168
HQ-29	5.674 ± 0.336	121.349 ± 1.648	2.123 ± 0.031	7.705 ± 1.002	6.798 ± 0.559	2.427 ± 0.162	1.950 ± 0.093	0.777 ± 0.051	148.803	4.727
HQ-30	6.894 ± 0.396	161.141 ± 1.124	4.967 ± 0.467	9.310 ± 0.778	11.823 ± 0.368	3.982 ± 0.303	1.637 ± 0.111	2.176 ± 0.158	201.929	9.321
HQ-31	3.801 ± 0.071	168.433 ± 1.873	4.563 ± 0.713	9.364 ± 1.608	5.525 ± 0.629	1.977 ± 0.139	2.026 ± 0.215	1.473 ± 0.170	197.163	6.340
HQ-32	3.812 ± 0.214	89.595 ± 0.874	1.748 ± 0.240	4.121 ± 0.616	6.055 ± 0.390	1.913 ± 0.208	1.609 ± 0.286	0.960 ± 0.086	109.813	3.991
HQ-33	4.604 ± 0.257	181.392 ± 1.143	2.803 ± 0.021	9.321 ± 1.822	8.744 ± 0.769	3.201 ± 0.314	1.119 ± 0.096	0.843 ± 0.076	212.026	7.329
HQ-34	7.278 ± 0.724	126.826 ± 0.803	2.786 ± 0.252	7.065 ± 0.050	6.408 ± 0.531	2.495 ± 0.239	1.141 ± 0.094	0.825 ± 0.073	154.824	5.073
HQ-35	1.886 ± 0.063	52.686 ± 0.230	1.337 ± 0.059	2.761 ± 0.163	1.531 ± 0.096	0.118 ± 0.074	0.667 ± 0.038	0.145 ± 0.004	61.132	2.288
HQ-36	6.013 ± 0.813	151.883 ± 0.593	2.980 ± 0.039	7.361 ± 0.050	7.965 ± 0.222	2.764 ± 0.020	1.645 ± 0.035	1.194 ± 0.067	181.806	5.955
HQ-37	11.949 ± 0.271	164.368 ± 0.447	3.043 ± 0.157	7.343 ± 0.064	4.234 ± 0.026	0.560 ± 0.033	2.555 ± 0.028	0.526 ± 0.093	194.577	6.822
HQ-38	8.417 ± 0.020	156.616 ± 0.386	4.173 ± 0.307	7.789 ± 0.232	15.992 ± 0.798	5.181 ± 0.241	2.576 ± 0.101	2.652 ± 0.123	203.397	11.395

^*∗*^TCEF: total content of the eight flavonoids.

**Table 4:** Average content of main flavonoid components in different growing areas (mg·g^−1^).

Flavonoids	Gansu	Inner Mongolia	Shandong	Shanxi	Henan	Hebei	Shaanxi
Scutellarin	6.695 ± 0.408	5.870 ± 0.186	6.011 ± 0.090	5.171 ± 0.145	5.300 ± 0.465	5.231 ± 0.148	7.066 ± 0.017
Baicalin	163.999 ± 0.26	131.090 ± 0.393	149.332 ± 0.654	132.058 ± 0.371	158.801 ± 0.453	132.604 ± 0.687	131.389 ± 0.142
Scutellarein	3.072 ± 0.156	2.958 ± 0.083	3.140 ± 0.299	2.592 ± 0.068	3.662 ± 0.161	2.446 ± 0.278	2.883 ± 0.093
Wogonoside	10.014 ± 0.442	6.410 ± 0.171	8.729 ± 0.874	6.778 ± 0.160	9.223 ± 0.511	6.836 ± 0.588	6.314 ± 0.261
Baicalein	15.449 ± 0.418	5.747 ± 0.149	7.995 ± 0.319	9.000 ± 0.378	8.234 ± 0.365	7.069 ± 0.231	7.431 ± 0.364
Wogonin	4.638 ± 0.184	1.801 ± 0.040	2.283 ± 0.123	2.678 ± 0.102	2.816 ± 0.224	2.536 ± 0.074	2.156 ± 0.176
Chrysin	2.340 ± 0.057	1.558 ± 0.091	1.817 ± 0.085	1.382 ± 0.069	1.660 ± 0.035	1.289 ± 0.072	1.861 ± 0.085
Oroxylin A	1.875 ± 0.061	1.151 ± 0.027	0.931 ± 0.073	1.131 ± 0.063	1.280 ± 0.027	0.876 ± 0.062	1.129 ± 0.076
TCEF	208.081	156.590	180.241	160.795	190.975	158.888	160.228
TF (%)	10.778	5.755	6.883	5.980	6.365	4.916	6.437

**Table 5:** The detection results of KMO and Bartlett.

Index		Value
KMO measure of sampling adequacy		0.587
Bartlett's test of sphericity	Approximate chi-square	30.447
	Freedom	10.000
	Significance	0.001

**Table 6:** Results of PCA of flavonoids.

Component	Initial eigenvalue			Extraction sum of squared loading		
	Sum	Variance (%)	Cumulative (%)	Sum	Variance (%)	Cumulative (%)
*X*_1_	3.574	71.477	71.477	3.574	71.477	71.477
*X*_2_	1.078	21.552	93.028	1.078	21.552	93.028
*X*_3_	0.323	6.453	99.482			
*X*_4_	0.019	0.375	99.857			
*X*_5_	0.007	0.143	100			

**Table 7:** Principal component matrix of flavonoids.

Component	Initial factor load		Score coefficient	
	1	2	1	2
*X*_1_	0.432	0.868	0.121	0.806
*X*_2_	0.906	−0.345	0.254	−0.320
*X*_3_	0.903	−0.383	0.253	−0.355
*X*_4_	0.915	0.065	0.256	0.060
*X*_5_	0.956	0.234	0.268	0.217

**Table 8:** Principal component scores of *Scutellaria baicalensis* from different origins.

Cultivated areas	*F*_1_ value	*F*_2_ value	*F* value	Ranking
Gansu	3.780	0.253	2.963	1
Inner Mongolia	−1.539	0.209	−1.135	6
Shandong	0.488	−0.146	0.341	2
Shanxi	−1.045	−0.232	−0.857	5
Henan	0.640	−0.801	0.307	3
Hebei	−1.572	−0.300	−1.277	7
Shaanxi	−0.752	1.017	−0.342	4

**Table 9:** Mean IC_50_ values for two free radicals and HepG2 from different growing areas.

Cultivated areas	IC_50_ (*μ*g/mL)		
	DPPH	ABTS	HepG2
Gansu	102.804 ± 0.202	35.540 ± 0.246	346.852 ± 3.612
Inner Mongolia	134.192 ± 0.084	55.600 ± 0.130	415.080 ± 4.183
Shandong	178.275 ± 0.056	89.140 ± 0.069	477.990 ± 4.362
Shanxi	211.619 ± 0.157	59.883 ± 0.120	408.828 ± 3.118
Henan	124.980 ± 0.310	57.850 ± 0.393	389.814 ± 2.119
Hebei	125.602 ± 0.539	39.022 ± 0.276	420.717 ± 4.478
Shaanxi	149.993 ± 0.187	47.328 ± 0.126	445.950 ± 2.712

## Data Availability

The data used to support the findings of this study are available from the corresponding author upon request.

## Supplementary Materials

Supplementary Materials The geographical and climatic information of different cultivated areas (Table S1) and the test results of sample recovery rate of 8 components (*n* = 6) (Table S2) are available in the Supplementary material document.
