# Supplementary material for: Comparative Study of the Flavonoid Content in Radix Scutellaria from Different Cultivation Areas in China
Source: Int J Anal Chem. 2023 Feb 10;2023:3754549. doi: 10.1155/2023/3754549 (PMC9938789; doi:10.1155/2023/3754549)
Supplement: Supplementary Materials — The geographical and climatic information of different cultivated areas (Table S1) and the test results of sample recovery rate of 8 components (n = 6) (Table S2) are available in the Supplementary material document. [file 3754549.f1.docx]

**T_ABLE_ S1** Geographical and climatic information of different cultivated areas.

| Cultivated areas | Longitude | Latitude | AMSL^*^ (m) | Mean Annual Temperature (℃) | Average Relative Humidity (%) | Mean Sunshine (h) | Annual Precipitation (mm) |
| --- | --- | --- | --- | --- | --- | --- | --- |
| Gansu | 103°41′29″ | 34°07′34″ | 2305.0 | 5.5 | 68.0 | 2214.9 | 635.0 |
| Inner Mongolia | 97°12′ | 37°24′ | 2067.0 | 7.0 | 71.0 | 3100.0 | 381.0 |
| Shandong | 114°47′30″ | 34°22′54″ | 1532.7 | 13.0 | 70.0 | 2290.0 | 676.5 |
| Shanxi | 110°14′ | 34°34′ | 1500.0 | 4.2 | 61.0 | 2331.0 | 358.0 |
| Henan | 110°21' | 31°23' | 2413.8 | 10.5 | 60.0 | 1285.7 | 407.7 |
| Hebei | 113°27′ | 36°05′ | 1200.0 | 8.5 | 65.0 | 2303.1 | 484.5 |
| Shaanxi | 105°29′ | 31°42′ | 1788.0 | 9.0 | 59.8 | 1851.9 | 390.7 |

*AMSL: Above mean sea level.

**T_ABLE_ S2** Test results of sample recovery rate of 8 components (*n*=6).

| Compounds | Sample /g | Original /mg | Spiked  /mg | Detected /mg | Recovery (%) | Average  recovery (%) | RSD(%) |
| --- | --- | --- | --- | --- | --- | --- | --- |
| scutellarin | 0.1002 | 0.677 | 0.542 | 1.225 | 101.107 | 99.066 | 1.344 |
|  | 0.1001 | 0.676 | 0.542 | 1.213 | 99.070 |  |  |
|  | 0.1003 | 0.678 | 0.677 | 1.356 | 100.148 |  |  |
|  | 0.1002 | 0.677 | 0.677 | 1.343 | 98.375 |  |  |
|  | 0.1005 | 0.679 | 0.812 | 1.475 | 98.030 |  |  |
|  | 0.1003 | 0.678 | 0.812 | 1.471 | 97.660 |  |  |
| baicalin | 0.1002 | 1.212 | 0.971 | 2.176 | 99.279 | 99.524 | 0.619 |
|  | 0.1003 | 1.213 | 0.971 | 2.173 | 98.867 |  |  |
|  | 0.1004 | 1.214 | 1.214 | 2.429 | 100.082 |  |  |
|  | 0.1002 | 1.212 | 1.214 | 2.412 | 98.847 |  |  |
|  | 0.1003 | 1.213 | 1.457 | 2.667 | 99.794 |  |  |
|  | 0.1002 | 1.212 | 1.457 | 2.673 | 100.275 |  |  |
| scutellarein | 0.1003 | 0.261 | 0.209 | 0.465 | 97.608 | 99.111 | 1.5 |
|  | 0.1002 | 0.261 | 0.209 | 0.474 | 101.914 |  |  |
|  | 0.1005 | 0.262 | 0.261 | 0.519 | 98.467 |  |  |
|  | 0.1006 | 0.262 | 0.261 | 0.521 | 99.234 |  |  |
|  | 0.1001 | 0.261 | 0.313 | 0.569 | 98.403 |  |  |
|  | 0.1002 | 0.261 | 0.313 | 0.571 | 99.042 |  |  |
| wogonoside | 0.1003 | 0.703 | 0.563 | 1.262 | 99.290 | 99.998 | 1.337 |
|  | 0.1004 | 0.704 | 0.563 | 1.258 | 98.401 |  |  |
|  | 0.1005 | 0.704 | 0.704 | 1.415 | 100.994 |  |  |
|  | 0.1001 | 0.702 | 0.704 | 1.421 | 102.131 |  |  |
|  | 0.1007 | 0.706 | 0.845 | 1.548 | 99.645 |  |  |
|  | 0.1002 | 0.702 | 0.845 | 1.543 | 99.527 |  |  |
| baicalein | 0.1003 | 0.603 | 0.482 | 1.082 | 99.378 | 100.433 | 1.188 |
|  | 0.1002 | 0.602 | 0.482 | 1.077 | 98.548 |  |  |
|  | 0.1007 | 0.605 | 0.603 | 1.216 | 101.327 |  |  |
|  | 0.1001 | 0.602 | 0.603 | 1.211 | 100.995 |  |  |
|  | 0.1005 | 0.604 | 0.724 | 1.339 | 101.519 |  |  |
|  | 0.1002 | 0.602 | 0.724 | 1.332 | 100.829 |  |  |
| wogonin | 0.1002 | 0.233 | 0.186 | 0.415 | 97.849 | 99.198 | 1.763 |
|  | 0.1004 | 0.233 | 0.186 | 0.421 | 101.075 |  |  |
|  | 0.1007 | 0.234 | 0.232 | 0.467 | 100.431 |  |  |
|  | 0.1005 | 0.234 | 0.232 | 0.458 | 96.552 |  |  |
|  | 0.1003 | 0.233 | 0.278 | 0.508 | 98.921 |  |  |
|  | 0.1003 | 0.233 | 0.278 | 0.512 | 100.360 |  |  |
| chrysin | 0.1004 | 0.107 | 0.086 | 0.191 | 97.674 | 99.183 | 1.439 |
|  | 0.1003 | 0.107 | 0.086 | 0.192 | 98.837 |  |  |
|  | 0.1001 | 0.107 | 0.107 | 0.216 | 101.869 |  |  |
|  | 0.1000 | 0.107 | 0.107 | 0.213 | 99.065 |  |  |
|  | 0.1002 | 0.107 | 0.128 | 0.234 | 99.219 |  |  |
|  | 0.1001 | 0.107 | 0.128 | 0.233 | 98.438 |  |  |
| oroxylin A | 0.1003 | 0.077 | 0.062 | 0.139 | 100.00 | 99.731 | 1.261 |
|  | 0.1002 | 0.077 | 0.062 | 0.138 | 98.387 |  |  |
|  | 0.1007 | 0.077 | 0.077 | 0.153 | 98.701 |  |  |
|  | 0.1003 | 0.077 | 0.077 | 0.155 | 101.299 |  |  |
|  | 0.1006 | 0.077 | 0.092 | 0.168 | 98.913 |  |  |
|  | 0.1005 | 0.077 | 0.092 | 0.170 | 101.087 |  |  |
